# Supplementary material for: Efficiency of protein synthesis inhibition depends on tRNA and codon compositions
Source: PLoS Comput Biol. 2019 Aug 1;15(8):e1006979. doi: 10.1371/journal.pcbi.1006979 (PMC6692046; doi:10.1371/journal.pcbi.1006979)
Supplement: S1 Text — (PDF) [file pcbi.1006979.s001.pdf]

Supplemental Information:

Efficiency of protein synthesis inhibition depends on tRNA and codon compositions

Sophia Rudorf

Theory and Bio-Systems, Max Planck Institute of Colloids and Interfaces, Potsdam, Germany

Contents

1 Rescaling measured growth rate into overall elongation rate for *E. coli* 1

2 Theoretical framework of translation elongation 1

2.1 Special Cases 5

2.2 Onset of translation at EF-Tu concentration $\mathcal{E}^*$ 7

1 Rescaling measured growth rate into overall elongation rate for *E. coli*

First, experimental data were manually extracted from Fig. 3 in Ref. [1] by using the software WebPlot-Digitizer [2], see Fig. S1 A). Second, experimental data from Table 2 ("Peptide chain elongation rate") by Liang et al. [3] were plotted and interpolated using Mathematica's function `Interpolation` [4]. The resulting interpolation function allows the conversion of data on specific growth rates into corresponding overall elongation rates, see Fig. S1 B).

2 Theoretical framework of translation elongation

Our analysis is based on the theoretical framework of translation developed in Refs. [5, 6]. Briefly, translation is described as a continuous-time Markov process to capture its stochastic nature. Based on biochemical studies of the ribosome [7–13], twelve ribosomal states are defined for each sense codon c in the

ribosomal A site. The different states, which are numbered from 0 to 11 as shown in Fig. S2, correspond to the binding of different ternary complexes to the ribosome and the different conformations of the ribosome/tRNA complex, respectively.

The ribosome dwells with an empty A site on a codon c in state $(c|0)$, until it binds a ternary complex that may be cognate, near-cognate, or non-cognate to codon c. Initial binding of a non-cognate ternary complex leads to state $(c|11)$, from which the ternary complex unbinds again, so that the ribosome returns back to state $(c|0)$. If a cognate ternary complex binds, the ribosome is in state $(c|1)$. From here, the cognate ternary complex can dissociate or move further into the A site, thus attaining the codon recognition state $(c|2)$. The recognition of a cognate ternary complex is followed by GTPase activation and GTP hydrolysis, leaving the ribosome in state $(c|3)$. The subsequent irreversible transition to state $(c|4)$ describes phosphate release and conformational rearrangements of EF-Tu. The cognate ternary complex is then usually fully accommodated in the A site, corresponding to a transition to state $(c|5)$. Alternatively, it may be released from the A site via the less probable transition back to state $(c|0)$. Binding of a near-cognate ternary complex is described by states $(c|6)$ to $(c|10)$. Recognized near-cognate ternary complexes are rejected by the ribosome via a backward transition from state $(c|7)$ to state $(c|6)$. In rare cases only, a near-cognate ternary complex will lead to GTPase activation and GTP hydrolysis followed by phosphate release and conformational rearrangements of EF-Tu via transitions to state $(c|8)$ and state $(c|9)$. If so, the near-cognate ternary complex will most likely be released, leading back to state $(c|0)$. With low probability, a near-cognate aa-tRNA gets fully accommodated in the A site via transition to state $(c|10)$. Finally, after a cognate or near-cognate tRNA has been fully accommodated in the states $(c|5)$ or $(c|10)$, the ribosome/tRNA complex undergoes the transition to the new state $(c'|0)$ at the next codon c', which describes the combined process of peptide bond formation and translocation.

Transitions between two states $(c|i)$ and $(c|j)$ occur with transition rates ω_{ij}. Numerical values for the *in-vivo* transitions rates are given in Ref. [6] and were obtained by minimizing the kinetic distance to the measured *in-vitro* rates as introduced in Ref. [5].

We assume that all transition rates are codon-independent, except for the binding rates ω_{01}, ω_{06}, and $\omega_{0,11}$ of cognate, near-cognate, and non-cognate ternary complexes, respectively. These rates are taken to be proportional to the concentrations $\hat{\mathrm{X}}_a$ of free cognate, near-cognate, and non-cognate ternary complexes as given by

$$\omega_{01} = \kappa_{\mathrm{on}} \sum_{a \in \mathbf{A}_{\mathrm{co}}(c)} \hat{\mathrm{X}}_a \,, \tag{1}$$

$$\omega_{06} = \kappa_{\mathrm{on}} \sum_{a \in \mathbf{A}_{\mathrm{nr}}(c)} \hat{\mathrm{X}}_a \,, \tag{2}$$

and

$$\omega_{0,11} = \kappa_{\mathrm{on}} \sum_{a \in \mathbf{A}_{\mathrm{no}}(c)} \hat{\mathrm{X}}_a \,, \tag{3}$$

where a indicates the tRNA species contained in a ternary complex, and $\mathbf{A}_{\mathrm{co}}(c)$, $\mathbf{A}_{\mathrm{nr}}(c)$, and $\mathbf{A}_{\mathrm{no}}(c)$ denote the sets of tRNA species that are cognate, near-cognate, and non-cognate to codon c, respectively. The binding rate constant κ_{on} is assumed to be identical for all cognate, near-cognate, and non-cognate ternary complexes, based on experimental findings [14].

The codon-specific elongation time $\mathrm{t}_{c,\mathrm{elo}}$ is the average time that a ribosome needs to finish a complete elongation cycle on codon c, i.e., to translate codon c. The inverse of the codon-specific elongating time is the codon-specific elongation rate $\omega_{c,\mathrm{elo}}$. The codon-specific elongation time $\mathrm{t}_{c,\mathrm{elo}}$ can be expressed by the sum of the average dwell times $\mathrm{t}_{(c|i)}$ that a ribosome spends in average per elongation cycle on codon c in the states $(c|i)$ with $i = 0, 1, \ldots, 11$, i.e.,

$$\omega_{c,\mathrm{elo}} \equiv \mathrm{t}_{c,\mathrm{elo}}^{-1} \equiv \left(\sum_{i=0}^{11} \mathrm{t}_{(c|i)} \right)^{-1} . \tag{4}$$

Analytical expressions for all dwell times $\mathrm{t}_{(c|i)}$ in terms of the concentrations $\hat{\mathrm{X}}_a$ of free ternary complexes and the transition rates ω_{ij} are given in Ref. [6]. However, it is important to note that, as mentioned above, the binding rates ω_{01}, ω_{06}, and $\omega_{0,11}$ depend on the concentrations of free cognate, near-cognate, and non-cognate ternary complexes. Therefore, the dwell times $\mathrm{t}_{(c|i)}$ depend on these concentrations as well.

Averaging the codon-specific elongation times over all codons in the "coding transcriptome", i.e., the population of all mRNA molecules in the cell, reveals the average elongation time per codon $\langle \mathrm{t}_{c,\mathrm{elo}} \rangle$. The overall elongation rate ω_{elo} is then given by the inverse of the average elongation time per codon $\langle \mathrm{t}_{c,\mathrm{elo}} \rangle$, i.e.,

$$\omega_{\mathrm{elo}} \equiv \langle \mathrm{t}_{c,\mathrm{elo}} \rangle^{-1} = \left(\sum_{c=1}^{61} \mathrm{p}_c \, \mathrm{t}_{c,\mathrm{elo}} \right)^{-1} , \tag{5}$$

where p_c denotes the normalized codon usage of codon c, which represents the probability that a randomly chosen sense codon in the mRNA population is equal to c. Numerical values of the codon usages in *E. coli* are given in Ref. [6]. The overall elongating rate ω_{elo} has been determined experimentally for *E. coli* under various growth conditions [3].

The overall elongation rate ω_{elo} depends on the concentrations of all species of free ternary complexes via the concentration-dependent binding of ternary complexes to the ribosome. The concentrations of

free ternary complexes are determined by the abundance of their components and the kinetics of the tRNA cycle depicted in Fig. 1 in the main text: tRNAs that have been used by translating ribosomes get translocated to the E sites from where they leave the ribosomes. Afterwards, the tRNAs bind to aminoacyl-tRNA synthetases that recharge them with their cognate amino acids. Finally, the recharged aa-tRNAs bind to EF-Tu molecules to form new ternary complexes that can bind to translating ribosomes. In *E. coli*, the total concentrations of tRNA molecules have been measured under various growth conditions [15]. However, due to the complex tRNA cycle, it is necessary to distinguish the total concentration of a tRNA molecule from the concentration of its corresponding ternary complex [6]. Recently, we have shown how the steady state concentration $\hat{\mathrm{X}}_b$ of free ternary complexes of species b can be calculated from the total concentration X_b of tRNA molecules of species b [6]. The concentration $\hat{\mathrm{X}}_b$ of free ternary complexes of species b depends on the concentrations $\hat{\mathrm{X}}_a$ of all ternary complex species, the concentration $\mathcal{E}^{\mathrm{fr}}$ of free EF-Tu molecules, the concentration $\mathcal{R}$ of ribosomes, the codon-dependent probabilities $\mathcal{P}_{c,\mathrm{co}}$ and $\mathcal{P}_{c,\mathrm{nr}}$ of cognate and near-cognate aa-tRNA accommodation defined in Ref. [6], and the codon usages p_c by

$$\begin{aligned}\hat{\mathrm{X}}_b = \mathrm{X}_b \Bigg(1 &+ \frac{\omega^{\mathrm{dis}}}{\kappa^{\mathrm{ass}}\,\mathcal{E}^{\mathrm{fr}}} \\ &+ \mathcal{R}\Bigg(\Phi_{\mathrm{co}} \sum_{c\in\mathbf{C}_{\mathrm{co}}(b)} \frac{\mathcal{P}_{c,\mathrm{co}}\,\mathrm{p}_c}{\sum\limits_{a\in\mathbf{A}_{\mathrm{co}}(c)} \hat{\mathrm{X}}_a} + \Phi_{\mathrm{nr}} \sum_{c\in\mathbf{C}_{\mathrm{nr}}(b)} \frac{\mathcal{P}_{c,\mathrm{nr}}\,\mathrm{p}_c}{\sum\limits_{a\in\mathbf{A}_{\mathrm{nr}}(c)} \hat{\mathrm{X}}_a} \\ &+ \omega_{\mathrm{elo}}\tau_{\mathrm{no}} \sum_{c\in\mathbf{C}_{\mathrm{no}}(b)} \frac{\mathcal{P}_{c,\mathrm{co}}\,\mathrm{p}_c}{\sum\limits_{a\in\mathbf{A}_{\mathrm{co}}(c)} \hat{\mathrm{X}}_a} \Bigg)\Bigg)^{-1} ,\end{aligned} \tag{6}$$

with the binding rate constant κ^{ass} and the dissociation rate ω^{dis}, which govern ternary complex formation from free EF-Tu molecules and aa-tRNAs. Under the assumption that E-site tRNAs leave the ribosome after a new aa-tRNA has bound to the A site, the dimensionless constants Φ_{co} and Φ_{nr} assume the values

$$\Phi_{\mathrm{co}} \equiv 2 + \omega_{\mathrm{elo}} \left(\tau_{\mathrm{co}} + \frac{1}{\omega^{\mathrm{re}}} + \frac{1}{\kappa^{\mathrm{ass}}\,\mathcal{E}^{\mathrm{fr}}} \right) , \tag{7}$$

$$\Phi_{\mathrm{nr}} \equiv 2 + \omega_{\mathrm{elo}} \left(\tau_{\mathrm{nr}} + \frac{1}{\omega^{\mathrm{re}}} + \frac{1}{\kappa^{\mathrm{ass}}\,\mathcal{E}^{\mathrm{fr}}} \right) , \tag{8}$$

where ω^{re} is the rate governing the recharging of de-aminoacylated tRNAs by synthetases with new amino acids. The constant time scales τ_{co}, τ_{nr}, and τ_{no} are given by

$$\tau_{\mathrm{co}} = \frac{1}{\omega_{\mathrm{rec}}\pi_{23}\pi_{45}} + \frac{1}{\omega_{23}\pi_{45}} + \frac{1}{\omega_{\mathrm{con}}\pi_{45}} + \frac{1}{\omega_{45}}\,, \tag{9}$$

$$\tau_{\mathrm{nr}} = \frac{1}{\omega_{\mathrm{rec}}\pi_{78}\pi_{9,10}} + \frac{1}{\omega_{78}\pi_{9,10}} + \frac{1}{\omega_{\mathrm{con}}\pi_{9,10}} + \frac{1}{\omega_{9,10}}\,, \tag{10}$$

$$\tau_{\mathrm{no}} = \frac{1}{\omega_{\mathrm{rec}}\pi_{23}\pi_{45}} + \frac{1}{\omega_{\mathrm{off}}\pi_{45}}\,, \tag{11}$$

where π_{ij} represents the probability of transition from state $(c|i)$ to state $(c|j)$ as given by

$$\pi_{ij} = \frac{\omega_{ij}}{\sum_k \omega_{ik}}\,. \tag{12}$$

For a detailed derivation of eq. (6), the reader is referred to Ref. [6].

2.1 Special Cases

1C-1T translation system. The following set of equations describes the dependence of the concentration $\hat{\mathrm{X}}$ of free ternary complexes as well as the overall elongation rate $\omega^{\mathrm{I}}_{\mathrm{elo}}$ and the concentration $\mathcal{E}^{\mathrm{fr}}$ of free functional EF-Tu molecules on the total concentration $\mathcal{E}$ of functional EF-Tu in the 1C-1T translation system:

$$\hat{\mathrm{X}}(\mathcal{E}) = \frac{\mathrm{X} - \mathcal{R}\Phi_{\mathrm{co}}(\mathcal{E})}{1 + \frac{\omega^{\mathrm{dis}}}{\kappa^{\mathrm{ass}}}\frac{1}{\mathcal{E}^{\mathrm{fr}}(\mathcal{E})}}\,, \tag{13}$$

$$\omega^{\mathrm{I}}_{\mathrm{elo}}(\mathcal{E}) = \frac{\rho_{\mathrm{co}}\kappa_{\mathrm{on}}\hat{\mathrm{X}}(\mathcal{E})}{1 + \left(\tau_{\mathrm{co}} + \frac{1}{\omega_{\mathrm{pro}}}\right)\rho_{\mathrm{co}}\kappa_{\mathrm{on}}\hat{\mathrm{X}}(\mathcal{E})}\,, \tag{14}$$

and

$$\mathcal{E}^{\mathrm{fr}}(\mathcal{E}) = \frac{1}{2}\beta^{\mathrm{I}}(\mathcal{E}) + \sqrt{\frac{\beta^{\mathrm{I}}(\mathcal{E})^2}{4} + \frac{\omega^{\mathrm{dis}}}{\kappa^{\mathrm{ass}}}\hat{\mathrm{X}}(\mathcal{E}) + \frac{\omega_{\mathrm{elo}}(\mathcal{E})}{\kappa^{\mathrm{ass}}}\mathcal{R}}\,, \tag{15}$$

with

$$\beta^{\mathrm{I}}(\mathcal{E}) \equiv \mathcal{E} - \mathrm{X} + 2\mathcal{R} + \frac{\omega_{\mathrm{elo}}(\mathcal{E})}{\omega^{\mathrm{re}}}\mathcal{R}\,, \tag{16}$$

where the same definitions are used as above and the dimensionless constant $\rho_{\rm co}$ is determined by the transition probabilities of ribosomal translation defined in eq. (12)

$$\rho_{\rm co} \equiv \frac{\pi_{12}\pi_{23}\pi_{45}}{1-\pi_{12}\pi_{21}}\,. \tag{17}$$

2C-2T translation system. The following set of equations describes the dependence of the concentrations $\hat{\rm X}_1$ and $\hat{\rm X}_2$ of free ternary complexes containing tRNA species 1 and 2, respectively, as well as the overall elongation rate $\omega_{\rm elo}^{\rm II}$ and the concentration $\mathcal{E}^{\rm fr}$ of free functional EF-Tu molecules on the total concentration $\mathcal{E}$ of functional EF-Tu in the 2C-2T translation system:

$$\begin{aligned}
\hat{\rm X}_1(\mathcal{E}) = {\rm X}_1 \Bigg(1 &+ \frac{\omega^{\rm dis}}{\kappa^{\rm ass}\,\mathcal{E}^{\rm fr}(\mathcal{E})} \\
&+ {\rm p}_1 \frac{\rho_{\rm co}\mathcal{R}\Phi_{\rm co}(\mathcal{E})}{\rho_{\rm co}\hat{\rm X}_1(\mathcal{E}) + \rho_{\rm nr}\hat{\rm X}_2(\mathcal{E})} \\
&+ {\rm p}_2 \frac{\rho_{\rm nr}\mathcal{R}\Phi_{\rm nr}(\mathcal{E})}{\rho_{\rm nr}\hat{\rm X}_1(\mathcal{E}) + \rho_{\rm co}\hat{\rm X}_2(\mathcal{E})} \Bigg)^{-1},
\end{aligned} \tag{18}$$

$$\begin{aligned}
\hat{\rm X}_2(\mathcal{E}) = {\rm X}_2 \Bigg(1 &+ \frac{\omega^{\rm dis}}{\kappa^{\rm ass}\,\mathcal{E}^{\rm fr}(\mathcal{E})} \\
&+ {\rm p}_2 \frac{\rho_{\rm co}\mathcal{R}\Phi_{\rm co}(\mathcal{E})}{\rho_{\rm co}\hat{\rm X}_2(\mathcal{E}) + \rho_{\rm nr}\hat{\rm X}_1(\mathcal{E})}. \\
&+ {\rm p}_1 \frac{\rho_{\rm nr}\mathcal{R}\Phi_{\rm nr}(\mathcal{E})}{\rho_{\rm nr}\hat{\rm X}_2(\mathcal{E}) + \rho_{\rm co}\hat{\rm X}_1(\mathcal{E})} \Bigg)^{-1},
\end{aligned} \tag{19}$$

$$\begin{aligned}
\omega_{\rm elo}^{\rm II}(\mathcal{E}) = \quad & \Bigg({\rm p}_1 \frac{\tau_{\rm co}\rho_{\rm co}\hat{\rm X}_1(\mathcal{E}) + \tau_{\rm nr}\rho_{\rm nr}\hat{\rm X}_2(\mathcal{E})}{\rho_{\rm co}\hat{\rm X}_1(\mathcal{E}) + \rho_{\rm nr}\hat{\rm X}_2(\mathcal{E})} \\
&+ {\rm p}_2 \frac{\tau_{\rm co}\rho_{\rm co}\hat{\rm X}_2(\mathcal{E}) + \tau_{\rm nr}\rho_{\rm nr}\hat{\rm X}_1(\mathcal{E})}{\rho_{\rm co}\hat{\rm X}_2(\mathcal{E}) + \rho_{\rm nr}\hat{\rm X}_1(\mathcal{E})} \\
&+ \frac{1}{\kappa_{\rm on}} \left(\frac{{\rm p}_1}{\rho_{\rm co}\hat{\rm X}_1(\mathcal{E}) + \rho_{\rm nr}\hat{\rm X}_2(\mathcal{E})} + \frac{{\rm p}_2}{\rho_{\rm co}\hat{\rm X}_2(\mathcal{E}) + \rho_{\rm nr}\hat{\rm X}_1(\mathcal{E})} \right) \\
&+ \frac{1}{\omega_{\rm pro}} \Bigg)^{-1},
\end{aligned} \tag{20}$$

and

$$\mathcal{E}^{\rm fr}(\mathcal{E}) = \frac{1}{2}\beta^{\rm II}(\mathcal{E}) + \sqrt{\frac{\beta^{\rm II}(\mathcal{E})^2}{4} + \frac{\omega^{\rm dis}}{\kappa^{\rm ass}}\left(\hat{\rm X}_1(\mathcal{E}) + \hat{\rm X}_2(\mathcal{E})\right) + \frac{\omega_{\rm elo}(\mathcal{E})}{\kappa^{\rm ass}}\mathcal{R}}\,, \tag{21}$$

with

$$\beta^{\rm II}(\mathcal{E}) \equiv \mathcal{E} - {\rm X}_1 - {\rm X}_2 + 2\mathcal{R} + \frac{\omega_{\rm elo}(\mathcal{E})}{\omega^{\rm re}}\mathcal{R}\,, \tag{22}$$

where the dimensionless constant ρ_{nr} is determined by the transition probabilities of ribosomal translation defined in eq. (12)

$$\rho_{\mathrm{nr}} \equiv \frac{\pi_{67}\pi_{78}\pi_{9,10}}{1-\pi_{67}\pi_{76}}\,. \tag{23}$$

2.2 Onset of translation at EF-Tu concentration $\mathcal{E}^*$

To understand what determines the onset of translation at $\mathcal{E}^*$, we further simplify the 2C-2T system:

i) We neglect the possibility of near-cognate incorporation, i.e., $\rho_{\mathrm{nr}} = 0$.

ii) We neglect any transition that is not related to ternary complex formation, i.e., $\tau_{\mathrm{co}} = 0$, $\frac{1}{\omega_{\mathrm{pro}}} = 0$, $\frac{1}{\omega^{\mathrm{re}}} = 0$. From $\tau_{\mathrm{co}} = 0$ follows that the dimensionless constant $\rho_{\mathrm{co}} = 1$, see eqs. (9) and (17).

iii) We neglect ternary complex dissociation, i.e., $\omega^{\mathrm{dis}} = 0$.

For these simplifications, the set of equations (18) to (22) describing the 2C-2T systems becomes

$$\hat{\mathrm{X}}_1(\mathcal{E}) = \mathrm{X}_1\left(1+\mathrm{p}_1\frac{\mathcal{R}\left(2+\omega_{\mathrm{elo}}(\mathcal{E})/\left(\kappa^{\mathrm{ass}}\,\mathcal{E}^{\mathrm{fr}}(\mathcal{E})\right)\right)}{\hat{\mathrm{X}}_1(\mathcal{E})}\right)^{-1}, \tag{24}$$

$$\Rightarrow \frac{\hat{\mathrm{X}}_1(\mathcal{E})}{\mathrm{p}_1} = \frac{\mathrm{X}_1}{\mathrm{p}_1} - 2\mathcal{R} - \frac{\omega_{\mathrm{elo}}(\mathcal{E})}{\kappa^{\mathrm{ass}}\mathcal{E}^{\mathrm{fr}}(\mathcal{E})}\mathcal{R}\,, \tag{25}$$

$$\hat{\mathrm{X}}_2(\mathcal{E}) = \mathrm{X}_2\left(1+\mathrm{p}_2\frac{\mathcal{R}\left(2+\omega_{\mathrm{elo}}(\mathcal{E})/\left(\kappa^{\mathrm{ass}}\,\mathcal{E}^{\mathrm{fr}}(\mathcal{E})\right)\right)}{\hat{\mathrm{X}}_2(\mathcal{E})}\right)^{-1} \tag{26}$$

$$\Rightarrow \frac{\hat{\mathrm{X}}_2(\mathcal{E})}{\mathrm{p}_2} = \frac{\mathrm{X}_2}{\mathrm{p}_2} - 2\mathcal{R} - \frac{\omega_{\mathrm{elo}}(\mathcal{E})}{\kappa^{\mathrm{ass}}\mathcal{E}^{\mathrm{fr}}(\mathcal{E})}\mathcal{R}\,, \tag{27}$$

$$\omega_{\mathrm{elo}}^{\mathrm{II}}(\mathcal{E}) = \kappa_{\mathrm{on}}\left(\frac{\mathrm{p}_1}{\hat{\mathrm{X}}_1(\mathcal{E})}+\frac{\mathrm{p}_2}{\hat{\mathrm{X}}_2(\mathcal{E})}\right)^{-1}, \tag{28}$$

and

$$\mathcal{E}^{\mathrm{fr}}(\mathcal{E}) = \frac{1}{2}\beta^{\mathrm{II}}(\mathcal{E}) + \sqrt{\frac{\beta^{\mathrm{II}}(\mathcal{E})^2}{4}+\frac{\omega_{\mathrm{elo}}(\mathcal{E})}{\kappa^{\mathrm{ass}}}\mathcal{R}} \tag{29}$$

$$\Rightarrow \mathcal{E}^{\mathrm{fr}}(\mathcal{E})^2 - \beta^{\mathrm{II}}(\mathcal{E})\mathcal{E}^{\mathrm{fr}}(\mathcal{E}) - \frac{\omega_{\mathrm{elo}}(\mathcal{E})}{\kappa^{\mathrm{ass}}}\mathcal{R} = 0 \tag{30}$$

$$\Rightarrow \frac{\omega_{\mathrm{elo}}(\mathcal{E})}{\kappa^{\mathrm{ass}}\mathcal{E}^{\mathrm{fr}}(\mathcal{E})}\mathcal{R} = \mathcal{E}^{\mathrm{fr}}(\mathcal{E}) - \beta^{\mathrm{II}}(\mathcal{E}) \tag{31}$$

with

$$\beta^{\mathrm{II}}(\mathcal{E}) \equiv \mathcal{E} - \mathrm{X}_1 - \mathrm{X}_2 + 2\mathcal{R}\,. \tag{32}$$

Note that this system has a meaningful solution only if $\frac{\mathrm{X}_1}{\mathrm{p}_1} > 2\mathcal{R}$ and $\frac{\mathrm{X}_2}{\mathrm{p}_2} > 2\mathcal{R}$, see eqs. (25) and (27) with $\frac{\omega_{\mathrm{elo}}(\mathcal{E})}{\kappa^{\mathrm{ass}}\mathcal{E}^{\mathrm{fr}}(\mathcal{E})}\mathcal{R} \geq 0$.

From eqs. (25), (27), (31), and (32) follows

$$\frac{\hat{X}_1(\mathcal{E})}{p_1} = \mathcal{E} - \left(X_1 + X_2 - \frac{X_1}{p_1} \right) - \mathcal{E}^{\rm fr}(\mathcal{E}) \,, \tag{33}$$

$$\frac{\hat{X}_2(\mathcal{E})}{p_2} = \mathcal{E} - \left(X_1 + X_2 - \frac{X_2}{p_2} \right) - \mathcal{E}^{\rm fr}(\mathcal{E}) \,. \tag{34}$$

The concentrations $\hat{X}_1(\mathcal{E})$, $\hat{X}_2(\mathcal{E})$, and $\mathcal{E}^{\rm fr}(\mathcal{E})$ cannot assume negative values. Therefore, the simplified 2C-2T system discussed here has a physically meaningful solution only if the concentration $\mathcal{E}$ of EF-Tu molecules is larger than or equal to $X_1 + X_2 - \frac{X_1}{p_1} + \mathcal{E}^{\rm fr}(\mathcal{E})$ and $X_1 + X_2 - \frac{X_2}{p_2} + \mathcal{E}^{\rm fr}(\mathcal{E})$.
Now, assume that $\frac{X_1}{p_1} > \frac{X_2}{p_2}$: For $\mathcal{E} = \mathcal{E}^* \equiv X_1 + X_2 - \frac{X_2}{p_2}$, both the concentrations $\hat{X}_2(\mathcal{E}^*)$ of ternary complexes of species 2 and $\mathcal{E}^{\rm fr}(\mathcal{E}^*)$ of free EF-Tu molecules, respectively, must vanish, otherwise one of the two would become negative as follows from eq. (33). In contrast, the concentration $\hat{X}_1(\mathcal{E}^*)$ of ternary complexes of species 1 attains the finite value $\hat{X}_1(\mathcal{E}^*) = X_1 - \frac{p_1}{p_2} X_2 > 0$.
Therefore, for $\mathcal{E} \gtrsim \mathcal{E}^* \Rightarrow \frac{\hat{X}_1(\mathcal{E})}{p_1} \gg \frac{\hat{X}_2(\mathcal{E})}{p_2}$, and eq. (28) further simplifies to

$$\omega_{\rm elo}^{\rm II}(\mathcal{E}) \approx \kappa_{\rm on} \, \frac{\hat{X}_2(\mathcal{E})}{p_2} \,, \tag{35}$$

such that the overall rate $\omega_{\rm elo}^{\rm II}(\mathcal{E})$ of peptide synthesis also vanishes at $\mathcal{E} = \mathcal{E}^*$. Note that from eqs.(29), (32) with $\beta^{\rm II}(\mathcal{E}^*) = \mathcal{E}^* - X_1 - X_2 + 2\mathcal{R} = -\frac{X_2}{p_2} + 2\mathcal{R} < 0$, and (35) follows that the concentration $\mathcal{E}^{\rm fr}(\mathcal{E})$ of free EF-Tu molecules vanishes in a self-consistent manner at $\mathcal{E} = \mathcal{E}^*$, and

$$\begin{aligned} \mathcal{E}^{\rm fr}(\mathcal{E}) \approx \; & \frac{1}{2} \left(\mathcal{E} - \mathcal{E}^* - \frac{X_2}{p_2} + \left(2 - \frac{\kappa_{\rm on}}{\kappa^{\rm ass}} \right) \mathcal{R} \right) \\ & + \sqrt{ \frac{1}{4} \left(\mathcal{E} - \mathcal{E}^* - \frac{X_2}{p_2} + \left(2 - \frac{\kappa_{\rm on}}{\kappa^{\rm ass}} \right) \mathcal{R} \right)^2 + \frac{\kappa_{\rm on}}{\kappa^{\rm ass}} \mathcal{R} \left(\mathcal{E} - \mathcal{E}^* \right) } \end{aligned} \tag{36}$$

for $\frac{\hat{X}_1(\mathcal{E})}{p_1} \gg \frac{\hat{X}_2(\mathcal{E})}{p_2}$. Obviously, the simplified 2C-2T system does not have a meaningful solution for EF-Tu concentrations $\mathcal{E} < \mathcal{E}^*$, because the right hand side of eq. (36) becomes negative.

References

[1] Peter H. Van Der Meide, Erik Vijgenboom, Marcel Dicke, and Leendert Bosch. Regulation of the expression of tufA and tufB, the two genes coding for the elongation factor EF-Tu in Escherichia coli. *FEBS Letters*, 139(2):325 – 330, 1982.

[2] Ankit Rohatgi. Webplotdigitizer 4.1. URL `https://automeris.io/WebPlotDigitizer`.

[3] Sung-Tzu Liang, Ying-Chun Xu, Patrick P. Dennis, and Hans Bremer. mRNA composition and control of bacterial gene expression. *Journal of Bacteriology*, 182(11):3037–3044, 2000.

[4] Wolfram Research, Inc. Mathematica, Version 11.1, 2017.

[5] Sophia Rudorf, Michael Thommen, Marina V. Rodnina, and Reinhard Lipowsky. Deducing the kinetics of protein synthesis *in vivo* from the transition rates measured *in vitro*. *PLoS Computational Biology*, 10(10):e1003909, 2014.

[6] Sophia Rudorf and Reinhard Lipowsky. Protein Synthesis in E. coli: Dependence of Codon-Specific Elongation on tRNA Concentration and Codon Usage. *PLoS ONE*, 10(8):1–22, 08 2015.

[7] Marina V. Rodnina, Tillmann Pape, Rainer Fricke, Lothar Kuhn, and Wolfgang Wintermeyer. Initial binding of the elongation factor Tu·GTP·aminoacyl-tRNA complex preceding codon recognition on the ribosome. *Journal of Biological Chemistry*, 271(2):646–652, 1996.

[8] Tillmann Pape, Wolfgang Wintermeyer, and Marina V. Rodnina. Complete kinetic mechanism of elongation factor Tu-dependent binding of aminoacyl-tRNA to the A site of the E-coli ribosome. *EMBO Journal*, 17(24):7490–7497, 1998.

[9] Kirill B. Gromadski and Marina V. Rodnina. Kinetic determinants of high-fidelity tRNA discrimination on the ribosome. *Molecular Cell*, 13(2):191–200, 2004.

[10] Kirill B. Gromadski, Tina Daviter, and Marina V. Rodnina. A uniform response to mismatches in codon-anticodon complexes ensures ribosomal fidelity. *Molecular Cell*, 21:369–377, 2006.

[11] Ute Kothe and Marina V. Rodnina. Delayed release of inorganic phosphate from elongation factor Tu following GTP hydrolysis on the ribosome. *Biochemistry*, 45(42):12767–12774, 2006.

[12] Niels Fischer, Andrey L. Konevega, Wolfgang Wintermeyer, Marina V. Rodnina, and Holger Stark. Ribosome dynamics and tRNA movement by time-resolved electron cryomicroscopy. *Nature*, 466 (7304):329–333, 2010.

[13] Jörg Mittelstaet, Andrey L. Konevega, and Marina V. Rodnina. A kinetic safety gate controlling the delivery of unnatural amino acids to the ribosome. *Journal of the American Chemical Society*, 135:17031 – 17038, 2013.

[14] Ingo Wohlgemuth, Corinna Pohl, Jörg Mittelstaet, Andrey L. Konevega, and Marina V. Rodnina. Evolutionary optimization of speed and accuracy of decoding on the ribosome. *Philosophical Transactions of the Royal Society B: Biological Sciences*, 366(1580):2979–2986, 2011.

[15] Hengjiang Dong, Lars Nilsson, and Charles G. Kurland. Co-variation of tRNA abundance and codon usage in Escherichia coli at different growth rates. *Journal of Molecular Biology*, 260(5):649–663, 1996.
