# Supplementary figures and images for: Efficiency of protein synthesis inhibition depends on tRNA and codon compositions
Source: PLoS Comput Biol. 2019 Aug 1;15(8):e1006979. doi: 10.1371/journal.pcbi.1006979 (PMC6692046; doi:10.1371/journal.pcbi.1006979)

A)

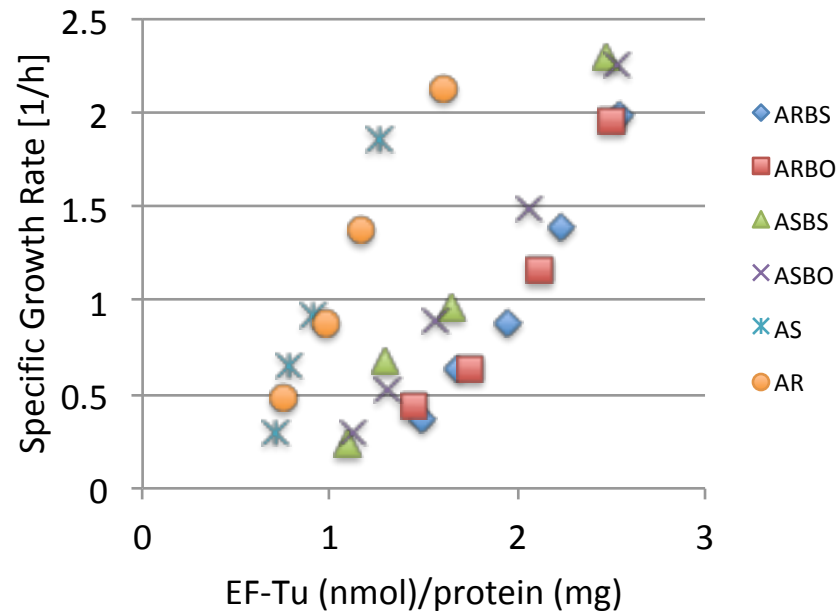

B)

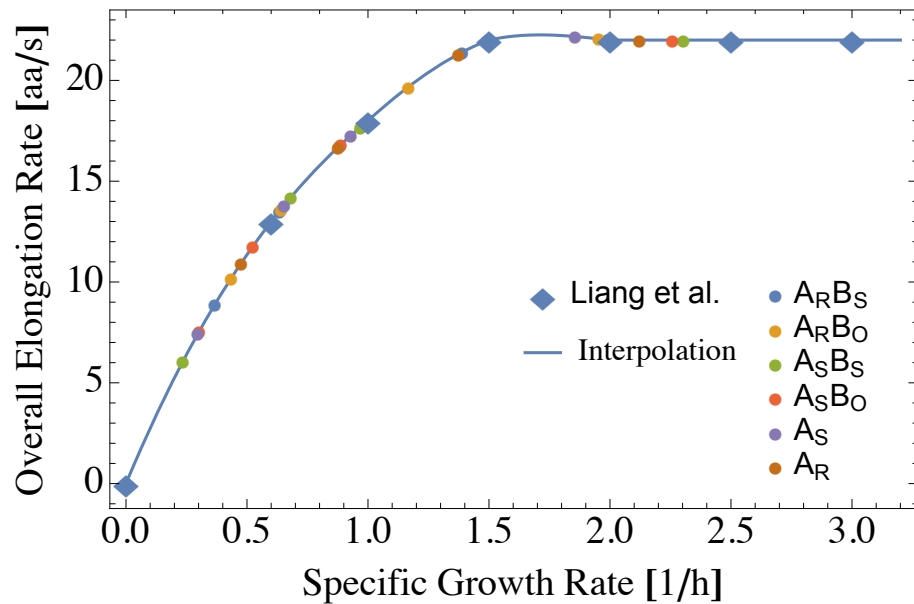

Supplement: S1 Fig — A) Growth rates and EF-Tu concentrations from van der Meide et al. (axes of ordinates and co-ordinates were interchanged) for different E. coli strains, see Table 2 and Fig. 3 in Ref. [30]. B) Interpolation of data from Liang et al. [42] (diamonds, solid line) relating growth rate and overall elongation rate in E. coli. Colored dots indicate growth rates as measured in A). (PDF) [file pcbi.1006979.s002.pdf]

*Non-Cognate*

*Near-Cognate*

*Cognate*

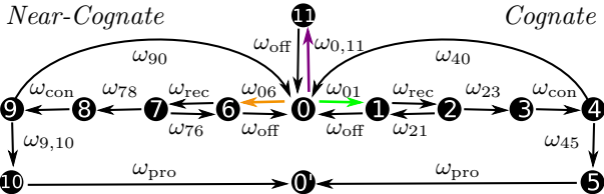

Supplement: S2 Fig — During translation of a codon c, the ribosome attains different states (c|i). Starting in state (c|0), it moves through the states of the cognate (i = 1…5) or near-cognate branch (i = 6…10), or attains state (c|11) upon binding of a non-cognate ternary complex. After translocation to the next codon c′, the ribosome reaches state (c′|0′). With the exception of the binding rates (of cognate, near-, and non-cognate ternary complexes; green, orange and purple arrows), all transition rates are assumed to be codon-independent. Figure previously published in [28]. (PDF) [file pcbi.1006979.s003.pdf]

A)

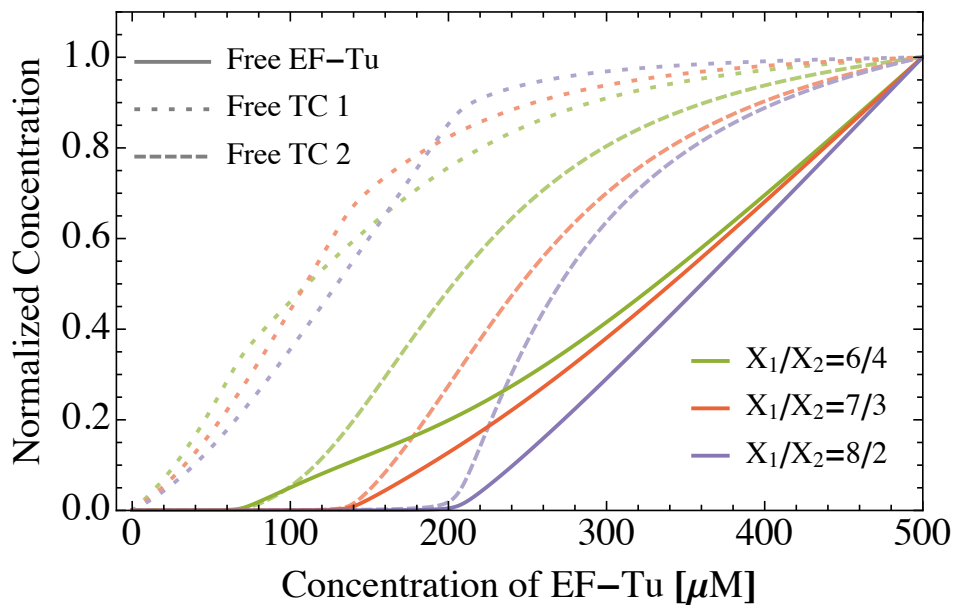

B)

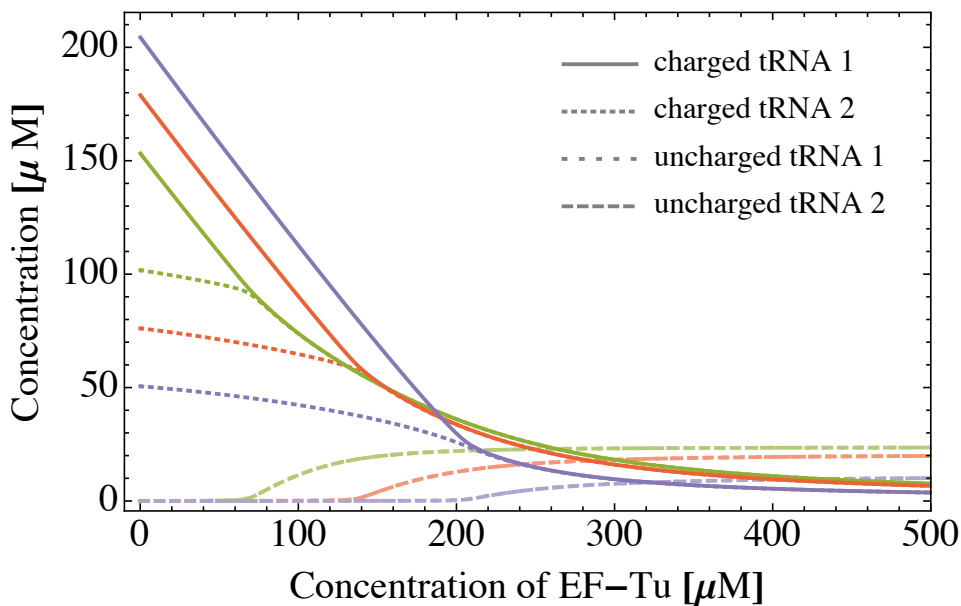

Supplement: S3 Fig — A) Normalized concentrations of free EF-Tu molecules (solid lines), free ternary complexes of the more abundant species 1 (dotted lines) and of the less abundant species 2 (dashed lines) as determined by the set of Eqs. (18)—(21) (p1/p2 = 1 and X1/X2 as indicated; concentrations relative to their value at 500 μM of EF-Tu). In the low-concentration regime E<E*, the free ternary complex concentration of the more abundant species 1 increases roughly linearly with E whereas the concentration of the less abundant species 2 remains practically zero up to E*. All parameters as in Fig 3B) in the main text. B) Concentrations of free, aminoacylated (charged) tRNAs and free, de-aminoacylated (uncharged) tRNAs of species 1 and 2, same parameters and corresponding color code as in A). Note that always a substantial fraction of tRNAs is bound to the A, P, and E sites of actively translating ribosomes, see [28] for detailed derivations of the corresponding equations. (PDF) [file pcbi.1006979.s004.pdf]

A)

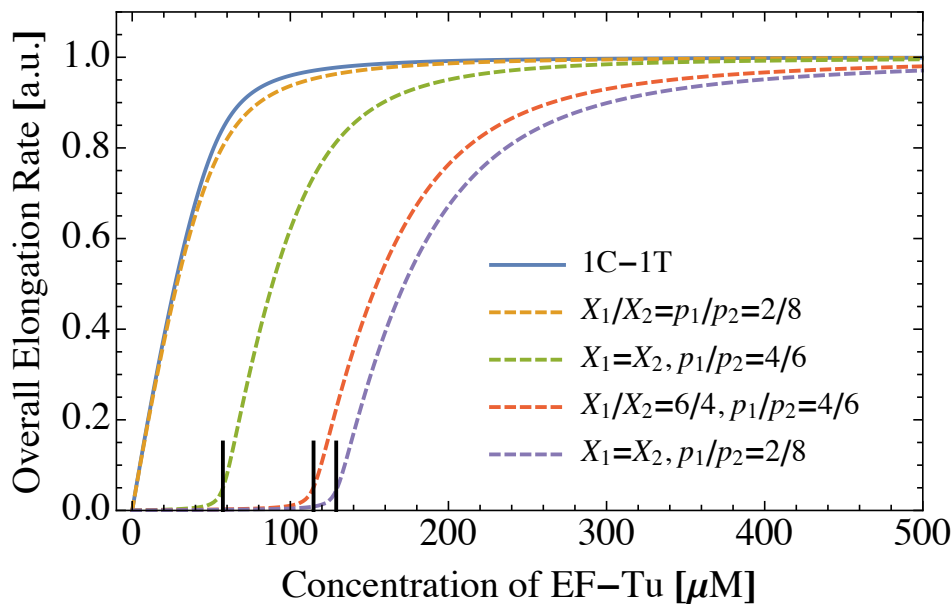

B)

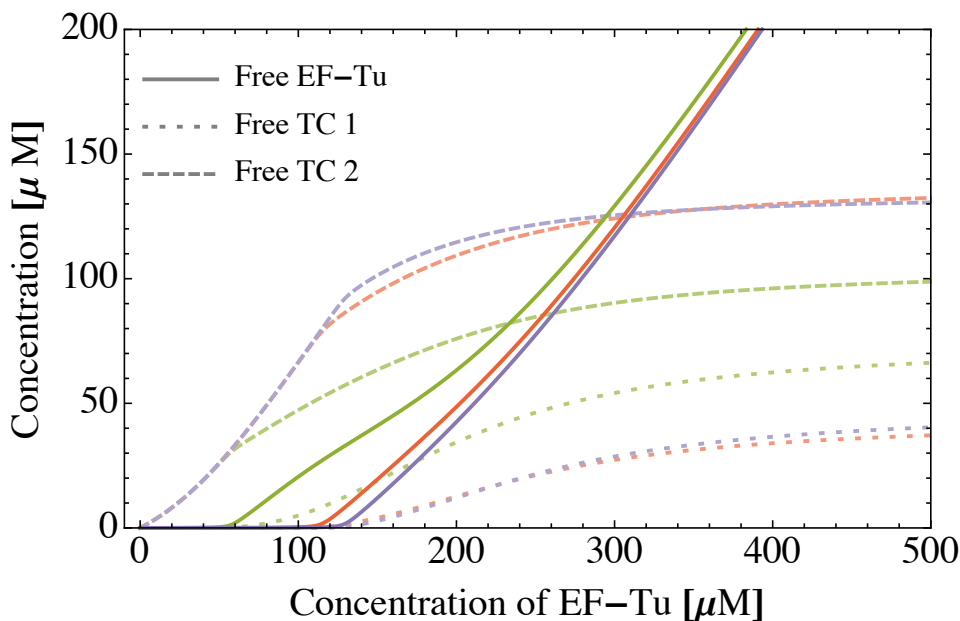

Supplement: S4 Fig — A) Overall elongation rate and B) concentrations of free EF-Tu molecules (solid lines), free ternary complexes of the more abundant species 1 (dotted lines) and of the less abundant species 2 (dashed lines) as a function of the concentration of EF-Tu for different codon usages pi and tRNA concentrations Xi (i = 1, 2) (see legend). All other parameters as in Fig 3A) and 3B) in the main text. Vertical solid black lines indicate EF-Tu threshold concentrations as given by Eq (1) in the main text. The same color code is used in A) and B). (PDF) [file pcbi.1006979.s005.pdf]

A)

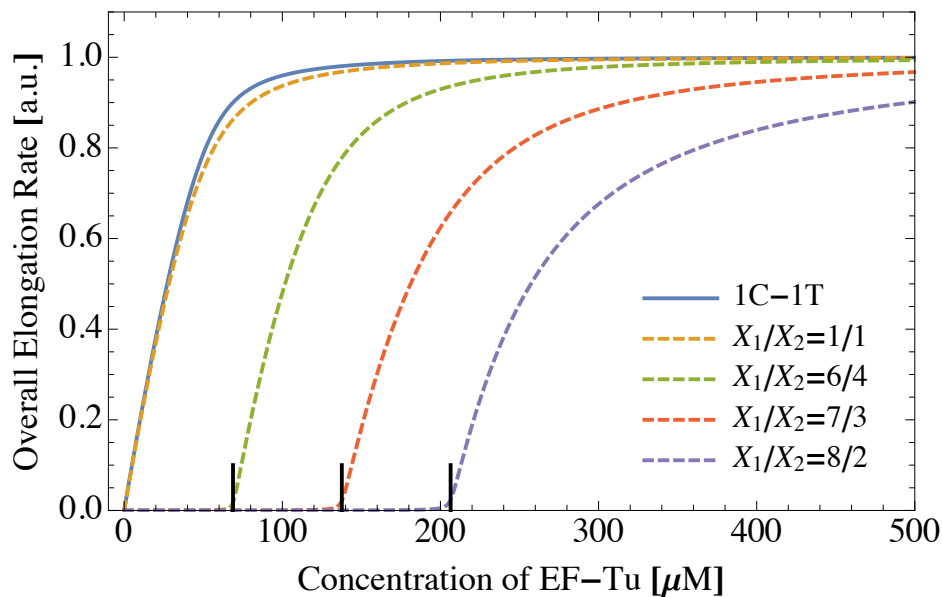

B)

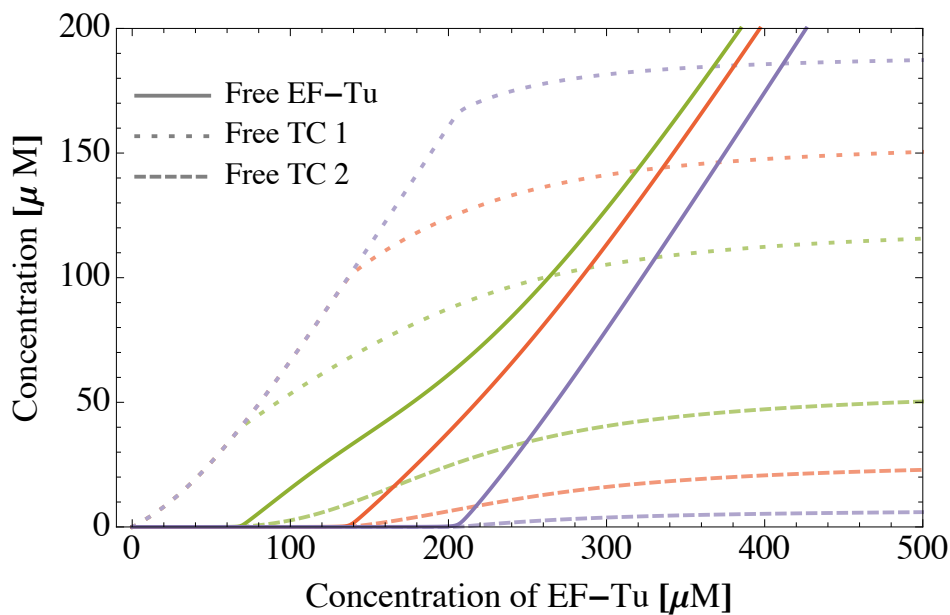

Supplement: S5 Fig — A) Overall elongation rate and B) concentrations of free EF-Tu molecules (solid lines), free ternary complexes of the more abundant species 1 (dotted lines) and of the less abundant species 2 (dashed lines) as a function of the concentration of EF-Tu. Here, the incorporation of near-cognate tRNAs is suppressed (ω9,10 = 0, see S2 Fig). All other parameters as in Fig 3A) and 3B) in the main text. (PDF) [file pcbi.1006979.s006.pdf]
